# Exploring women’s experiences during childbirth in health facilities during COVID-19 pandemic in occupied palestinian territory: a cross-sectional community survey

**DOI:** 10.1186/s12884-022-05265-y

**Published:** 2022-12-22

**Authors:** Niveen ME Abu-Rmeileh, Yasmeen Wahdan, Hedieh Mehrtash, Khitam Abu Hamad, Arein Awad, Ӧzge Tunçalp

**Affiliations:** 1 Institute of Community and Public Health, Birzeit University, Ramallah, West Bank, Occupied Palestinian territory, Palestine; 2 WHO/World Bank Special Programme of Research, Development and Research Training in Human Reproduction, Department of Sexual and Reproductive Health and Research, UNDP/UNFPA/UNICEF, World Health Organization, Geneva, Switzerland; 3 School of Public Health, Al-Quds University, Jerusalem, Occupied Palestinian territory, Palestine

**Keywords:** Mistreatment, Health facility, Quality of care, Palestine, COVID-19

## Abstract

**Introduction:**

This study aims to assess the prevalence of mistreatment during childbirth in the occupied Palestinian territory and to explore factors associated with mistreatment.

**Methods:**

A cross-sectional study of women who gave birth in the West Bank and Gaza Strip health facilities. The survey was administered over the phone to women up to 8 weeks post-partum. Data collection took place between July 2020 and March 2021.

**Results:**

A total of 745 women participated in the study, 36·25% were from the Gaza Strip and 63·75% from the West Bank. The prevalence of mistreatment was 18·8% in which women reported any verbal abuse, physical abuse, or stigma or discrimination during childbirth, with verbal abuse as the most common form of mistreatment reported. Physical abuse was more likely to be reported by women with no labour companion with them (OR: 3·11, 95%CI: 1·24 − 7·99). Verbal abuse was more likely to be reported by women with less than three live births (OR: 1·71, 95%CI: 1·06 − 2·76, women with no birth companion (OR: 2·72, 95%CI: 1·36 − 3·80) and more likely to be reported if curtains wre not used (OR: 2·55, 95%CI: 1·33 − 4·88). Women with less education were more likely to report long waiting times or delays in receiving services compared to women with higher education (OR: 1·40, 95%CI: 1·06 − 2·10).

**Conclusion:**

For the first time using the World Health Organisation (WHO) tool in the Eastern Mediterranean region, the study findings, show the occurrence of mistreatment and identify areas to be strengthened to ensure that all women have a respectful childbirth experience within health facilities.

**Supplementary Information:**

The online version contains supplementary material available at 10.1186/s12884-022-05265-y.

- **What is already known on this topic** – few studies informing the global and regional literature of women’s experiences of care in the Eastern Mediterranean region and specifically in a conflict setting such as the oPt.
- **What this study adds** – This study is based on a standardized validated tool that was developed and tested in four countries. The tool was further translated, adapted and tested using a rigorous process to Arabic. The study findings, show the occurrence of mistreatment and identify areas to be strengthened to ensure that all women have a respectful childbirth experience within health facilities.
- **How this study might affect research, practice, or policy** **–** The WHO mistreatment during childbirth tool can be used in the Eastern Mediterranean region which will allow for cross-country comparisons.

## Introduction

In the Eastern Mediterranean region, the maternal mortality ratio (MMR) has decreased from 283 to 100,000 live births in 1990 to 237 per 100,000 live births in 2015, with a 16% reduction. Variations and disparities in maternal mortality levels were observed between high-income and low-income countries in the region. MMR ranged from 6 deaths per 100,000 live births in Kuwait to 789 deaths per 100,000 live births in Afghanistan [1]. This variation in the achieved decline might be explained by the increase in the number of countries affected by conflict and humanitarian [2, 3] conditions within the region in addition to the recent COVID-19 pandemic, along with the significant difference in the economic status and the availability of health services [4].

Despite the observed decline in maternal mortality in the region, women suffer from problems beyond access to obstetric services. These range from routine use of unsafe and non-evidence-based practices to failure to take women’s preferences into account [5], and excessive levels of medical interventions [6, 7]. Still, there is limited information available about the quality of care during the childbirth process in the region. A recent scoping review of the EMR reported that the most common types of mistreatment during childbirth are physical abuse and non-dignified care [8]. In the past ten years, maternal health indicators have been improving in the occupied Palestinian territory (oPt). The fertility rate dropped from 4·5 in 2006 to 3·8 in 2019 [9]. Maternal mortality rate has declined steadily reaching 24·7 per 100,000live births in 2014 [10]. Almost all deliveries occur in health facilities (99·4%) and/or are attended by trained professionals (99·7%) [9]. Antenatal services are divided almost equally between governmental maternal and child health clinics and other private and non-governmental organizations clinics; however, several deliveries take place in crowded and understaffed governmental hospitals [11]. In terms of the health workforce, maternal health care providers work within a difficult and resource-constrained environment [12]. The unstable conditions and the acute emergency events in the oPt pose high-pressure on the health system in general and the medical staff working within the system [13]. The priority shifts to emergency conditions with questionable attention to routine /standard practices. The need to study the quality of reproductive health services was highlighted in a recent research priority exercise conducted in the oPt [14].

Mistreatment of women during facility-based childbirth is a significant public health issue and is gaining worldwide attention [15]. Mistreatment was reported with system distrust and avoidance of facility-based delivery care, affecting women’s health and health-seeking behavior. There remains a lack of agreement on measurement definitions of mistreatment worldwide including the Arab countries [2]. A recent systematic review was conducted to identify the types of mistreatment, terminology, and tools in the Arab world. The review revealed that very limited studies that focus on this issue, no unified definition, and no standardized methods. Studies identified focused more on positive experiences and women’s satisfaction [15, 16]. However, there needs to be more data and research on this topic in Eastern Mediterranean countries.

This study aimed to estimate the prevalence of mistreatment during childbirth in the oPt using WHO’s community survey tool and to explore individual, provider, institutional and health systems factors that either promote or prevent mistreatment of women during childbirth in health facilities.

## Methods

The study followed the methodology of the WHO multi-country study on how women are treated during facility-based childbirth [17]. The WHO multi-country study used two standardized measurement tools; continuous observations of women throughout labour and childbirth, and community-based surveys with women up to 8 weeks after childbirth. This study uses community-based survey approach to measure the prevalence of mistreatment in the oPt.

Five health facilities (three governmental hospitals, one private hospital, and one non-governmental hospital) were selected based on hospital characteristics. Hospitals were included if they were secondary-level Facilities or higher and had at least 200 births per month with a well-defined community catchment area. Four hospitals were in the West Bank (two from the Center, two from the South of the West Bank), and one from the Gaza Strip. The characteristics of the selected facilities are listed in the supplement file (Supplement file annex 1).

Women were included as survey participants if they were at least 18 years old, had been admitted for childbirth, were willing to provide written participation consent, and lived within 15 km of the selected hospital. Women were excluded if they were admitted for reasons other than childbirth, were a first-degree relative to a facility employee, were distressed or otherwise unable to reasonably provide informed consent, resided more than 15 km from the facility, or were unable to provide sufficient contact information.

The required sample size was 600 women across the five facilities, based on ± 5% precision, 80% sensitivity, 5% type 1 error (two-tailed), and 50% prevalence. We assumed a 25% loss to follow-up between recruitment. Based on the patient load and the number of births per month in each hospital aiming for a minimum of 730 women, we recruited 130 from each of the governmental hospitals in the West Bank and 100 from each of the private hospitals, in addition to 270 from Gaza hospital.

This study was approved by the WHO Ethical Review Committee, WHO Review Panel on Research Projects, the Institute of Community and Public Health at the Birzeit University Ethics Research Committee, and the Helsinki Committee of the Ministry of Health.

### Procedures

Each study site had one data collector to complete the recruitment process and another two research assistants to conduct the interviews. The data collectors were staff members working in each health facility from other departments that received specific training in safety measures and protection against COVID-19. For this study, they were trained on recruitment instructions, recruitment strategies, including the wording, identification, eligibility lists, and reporting of the participants. In addition, the data collectors were responsible for the screening log for community surveys in the hospital. Due to the Covid-19 lockdown and protective measures, the method of the interview from the WHO multi-country study was modified from a face-to-face interview to a telephone interview. Data collectors informed the participants that the interviews would be carried out through a virtual method and women had the choice to select the preferred method within two to four weeks of delivery.

The research assistants/interviewers were responsible for analysing the community survey screening logs in the hospital and determining woman’s eligibility. The survey was conducted by experienced research assistants who had been given adequate training in conducting surveys and, were not healthcare providers. These researchers had health backgrounds in pharmacy, dentistry, nutrition, and nursing. Furthermore, they also took part in training to obtain consistent results and improve the validity of the answers.

Oral informed consent was obtained from women at the hospital after delivery to contact them within two to six weeks postpartum. In addition, we conducted telephone interviews with women to reduce fieldworker mobility as it posed a high risk for the team and the community. The interviewers obtained a second oral informed consent from women after they agreed to participate in the study and subsequently conducted the telephone interviews. The interviewers attempted to contact women up to five times over a two-week period. Eligible women, whom we could not reach, were called up to five times before reporting the loss to follow-up. All interviews were conducted within two to six weeks postpartum.

### Survey tool

The methodological development of the community survey tool has been described in a previous study [18]. An Arabic adapted version of the tool was developed for the study. The original tool was written in English and translated it into Arabic (i.e. the local language in the oPt) using the following defined steps: The forward translation was done by two independent bilingual translators whose native language was Arabic. The backward translation included translating back the questionnaires from Arabic to English in order to resolve any inconsistencies and to ensure accurate translation It was performed by two independent bilingual translators whose native language was English. Before data collection, a focus group discussion (FGD), using a modified cognitive interview approach, was conducted to test the prefinal version with mothers who had recently given birth. Furthermore, during the FGD, the participants were asked to elaborate on what they understood from each question to ensure that the translated items were conceptually equivalent to the original ones and that the language used was appropriate and acceptable. Finally, experts conducted an independent review to finalise the translated tool [19]. The experts reviewed backward translation differences and determined whether the Arabic translated version was conceptually equivalent to the original version, and resolved any discrepancies. The final version of the translated tool took considered all comments resulting from the previous steps; hence, the tool was ready to be applied to a larger sample from the target population. The survey questions were well understood by women. However, some questions women reported as not applicable to the Palestinian context including perineal re-stitching, were kept in the study to assess whether there was a need to keep or remover them in future studies. The question on “staff suggested or asked the woman or companion for a bribe” was also modified in this context to include informal payment or gift.

The main questionnaire included details relating to socio-demographic data, and mistreatment typology (including physical & verbal abuse, stigma or discrimination) [20], and other sections including maternal outcome, baby outcome, labour management practices, interventions, post-partum depression, and overall care satisfaction. An additional section related to assessing health service needs and provision during the COVID-19 pandemic was included, with questions about the need and availability of health, counseling, and psychological services during the antenatal period and the need and availability of information about COVID-19, protective measures and COVID 19 infection at the health facility during the childbirth process.

Data were collected using a digital, tablet-based tool offered by KoBo ToolboX, between July 2020 and February 2021, and itwas reviewed on a daily basis during that period.

### Statistical analysis

Variable grouping and categorization followed the same procedure described by Bohren and colleagues [17]. The socio-demographic variables (age, education, marital status, number of pregnancies, number of previous births) and health outcome data (such as physical abuse, verbal abuse, stigma or discrimination, failure to meet a professional standards, vaginal examinations, pain relief) were categorised as both a proportion of the total study population and by region, West Bank and the Gaza Strip.

For the mistreatment typology, the different sub-items ‘physical or verbal abuse and stigma or discrimination’ were aggregated into dichotomous variables (yes or no responses), then aggregated into a single indicator for each item. Multivariable Logistic Regression models were applied to assess women’s characteristics associated with different forms of mistreatment (e.g. age, education, and gravidity). For specific models, the use of privacy measures (e.g. use of curtains) and companion presence at any time were also evaluated. The following domain/typology (physical abuse, verbal abuse, stigma or discrimination, non-consented vaginal examination, non-private vaginal examination, neglect, long wait times or delays, and mobilization) were studied in separate regression models. All measures and definitions are listed in the supplement file. Data analysis was subsequently conducted using SPSS software.

### Patients and public involvement

women were not directly involved in the study’s design. However, they were part of the tool validation and translation.

## Results

Recruitment took place between mid-February and March 5th, 2020. At that time, the team recruited around 220 women from Gaza and Hebron. The data collection was suspended due to the COVID-19 pandemic, restarted again in mid-July 2020, and completed end of February 2021. A total of 745 women participated in the study (270/745 (36·25%) from the Gaza Strip and 475/745 (63·75%) from the West Bank) across the five health facilities. Most women were between the ages of 20 and 34 (602/745, 80·8%). 44%(328/745) of women had post-secondary education with a slightly higher percentage among women from the West Bank. The percentage of women with four or more pregnancies was 37·6% and the percentage of women with one pregnancy was 23·9%. The percentage of women with four or more births was about 32% and the at of women with one birth was 27%. Finally, women with Caesarean births were about 27%, as shown in Table 1.

**Table 1:** Sociodemographic information and obstetric history

	**Gaza** **(*N*=270)**	**West Bank** **(*N*=475)**	**Total** **(*N*=745)**	***p*-value**
**Maternal age (years)**				
≤19	19(7·0%)	28(5·9%)	47(6·3%)	0·368
20-24	90(33·3%)	135(23·4%)	225(30·2%)	
25-29	81(30·0%)	148(31·2%)	229(30·7%)	
30-34	49(18·1%)	99(20·8%)	148(19·9%)	
≥ 35	31(11·5%)	65(13·7%)	96(12·9%)	
**Marital status**				
Currently married	268(99·3%)	474(99·8%)	742(99·6%)	0·299
Single^a^	2(0·7%)	1(0·2%)	3(0·4%)	
**Education**				
No education	0(0·0%)	1(0·3%)	1(0·3%)	0·001
Primary education	14(5·2%)	17(3·5%)	31(4·1%)	
Some secondary education	133(49·3%)	160(33·7%)	293(39·3%)	
Secondary education	22(8·1%)	65(13·7%)	87(11·7%)	
Tertiary education	100(37·0%)	228(48·0%)	328(44·0%)	
Vocational training	1(0·4%)	4(0·8%)	5(0·7%)	
**Number of pregnancies (gravidity)**				
1	74(27·4%)	104(21·9%)	178(23·9%)	0·029
2	44(16·3%)	115(24·2%)	159(21·3%)	
3	42(15·6%)	86(18·1%)	128(17·2%)	
4+	110(40·7%)	170(35·8%)	280(37·6%)	
**Number of previous births (parity)**				
1	79(29·4%)	121(25·6%)	200(27·0%)	0·010
2	48(17·8%)	119(25·2%)	167(22·5%)	
3	41(15·2%)	96(20·3%)	137(18·5%)	
4+	101(37·5%)	137(29·0%)	238(32·1%)	
**Mode of childbirth**				
Vaginal birth	202(74·8%)	340(71·6%)	542(72·8%)	0.001
Caesarean birth	68(25·2%)	135(28·4%)	203(27·2%)	

^a^Separated, divorced, or widowed

Table 2 presents the prevalence of reported mistreatment of women during childbirth using the typology of mistreatment. A total of 140/745 (18·8%) women reported physical abuse, verbal abuse or stigma or discrimination during childbirth with slight variation between the West Bank 78/475 (16·4%) and the Gaza Strip 62/270 (23·0%). Verbal abuse was reported by 124/745 (16·6%) women with slightly higher reports in the Gaza Strip 45/270 (20·0%) compared to the West Bank 70/475 (14·7%). The most common form of verbal abuse was being shouted at, reported by 65/745 (8·7%), and being hissed with 44/745 (5·9%). The percentage of women reporting physical abuse was 25/745 (3·4%); the most common form was forceful downward abdominal pressure 19/745 (2·6%). Only 5/745 (0·7%) women reported stigma or discrimination, mainly because of their age 4/5 (80%).

**Table 2:** Mistreatment of women during childbirth across five facilities in the oPt based on the community survey

	**Gaza**	**West Bank**	**Total**	***p*-value**
**Any physical abuse, verbal abuse, or stigma or discrimination**	62(23·0%)	78(16·4%)	140(18·8%)	0·019
Any physical abuse	14(5·2%)	11(2·3%)	25 (3·4%)	0·032
Any verbal abuse	54(20·0%)	70 (14·7%)	124(16·6)	0·041
Any stigma or discrimination	1(0·4%)	4(0·8%)	5(0·7%)	0·404
**Failure to meet professional standard**				
Caesarean section (N=745)	68(25·2%)	135(28·4%)	203(27·2%)	0·193
Non-consented Caesarean section (N=203)	25(36·7%)	41(30·3%)	65(32·1%)	0·32
Episiotomy^b^ (N=251)	103(38·0%)	148(57·5%)	251(33·6%)	0·373
Non-consented Episiotomy (N=251)	59(57·2%)	117(69·6%)	176(64·9%)	0·001
Induction of labour (N=700)	49(20·1%)	91(20·0%)	140(20%)	0·521
Non-consented Induction of labour (N=140)	41(83·7)	76(83·5%)	117(83·5%)	0·814
**Vaginal examination**				
Woman had any vaginal examination	228(84·0%)	402(84·6%)	630(84·4%)	0·521
Non-consented vaginal examination	118(51·7%)	269(66·9%)	387(61·4%)	0·002
Vaginal examination conducted privately	193(84·6%)	369(91·8%)	562(89·2%)	0·005
Discussed your health information privately	162(71·7%)	331(82·5%)	493(78·6%)	0·001
General description of experience of vaginal examinations				
Comfortable	87(38·2%)	91(22·7%)	178(28·3%)	
A little uncomfortable	91(39·9%)	157(38·9%)	248(39·3%)	<0·001
Quite uncomfortable	35(15·4%)	99(24·7%)	134(21·3%)	
Very uncomfortable	15(6·6%)	55(13·7%)	70(11·1%)	
**Pain relief**				
Woman not offered pain relief during time in hospital	198(73·6%)	280(59·2%)	487(64·4%)	<0·001
Woman requested pain relief	110(40·7%)	274(57·8%)	384(51·5%)	<0·001
Woman did not receive pain relief	98 (89·1%)	109(39·8%)	187(48·7%)	<0·001
Woman denied pain relief during time in hospital	59(22·3%)	53(11·2%)	112(15·2%)	<0·001
**Neglect and abandonment**				
No staff member present when the baby came out ^b^	15·0(7.6%)	40(11·9%)	55(10·1%)	0·074
Woman waited for long periods of time before attended by health workers	101(38·1%)	141(29·7%)	244(32·8%)	·011
Woman felt ignored, neglected, or that presence was a nuisance for health workers or staff	86(31·8%)	88(18·6%)	174(23·4%)	<0·001
**Communication**				
Woman felt that health workers or staff did not listen and respond to her concern	144(54·5%)	344(73·7%)	488(65·5%)	<0·001
**Supportive care**				
Woman not allowed to have labour companion during labour process	60(22·3%)	38(8·0%)	98(13·2%)	0·001
Companion not present at any time and birth	9(3·3%)	44(9·3%)	53(7·1%)	<0·001
**Autonomy**				
Woman did not have easy access to water or oral fluids during labour	121(48·6%)	149(33·5)	270(38·9%)	<0·001
Not allowed to eat ^b^)	9(4·5%0	21(6·2%)	30(5·6%)	0·271
Woman not told she could mobilise during labour, and did not mobilise during labour	105(39·2%)	115(24·2%)	220(29·6%)	0·389
Woman not allowed to deliver in her preferred position	2(12·5%)	8(35·3%)	8(24·2%)	0·186
Woman or baby detained in hospital because of inability to pay hospital bills	2(0·7%)	1(0·2%)	3(0·4%)	0·299
**Health systems**				
Woman instructed to clean up blood, urine, faeces, or amniotic fluid	8(2·6%)	28(5·7%)	36(4·7%)	0·036
Staff suggested or asked the woman or companion for a bribe, informal payment, or gift^a^	160(59·7%)	9(1·9%)	169(22·3%)	<0·001
Curtains, partitions, or other measures used to provide privacy for the woman throughout labour, childbirth, and post-partum periods	251(93·7%)	440(92·6%)	691(93·0%)	0·357

^a^Asking for a gift for the celebration

^b^Number of women with vaginal birth, *N*=542

203/745 (27·2%) women had a caesarean section, of which 65/203 (32·1%) have not consented. 140/700 (20%) women had induction of labour, of which 117/140 (83·5%) have not consented. Most women 630/745 (84·4%) had any vaginal examination (at least one); of women who received a vaginal examination (630), 387/630 (61·4%) have not consented; and, 562/630 (89·2%) of women reported that vaginal examination was conducted privately. A high percentage of women reported having vaginal examinations conducted privately 369/402 (91·8%) and their related health information was discussed privately 193/228 (82·5%) in the West Bank. 562/630 (84·5%) women reported having vaginal exams conducted privately and 493/627(71·7%) health information discussed privately in the Gaza Strip. 487/745 (65·4%) were not offered pain relief while in the hospital. A total of 384/745 (51·5%) of women requested pain relief and 187/384 (48·7%) did not receive it, with higher percentages of women in the Gaza Strip compared to the West Bank. 174/745 (23·4%) of women felt ignored or neglected, mainly in the Gaza Strip 86/270 (31·8%). Further, 488/745(65·5%) of women reported that they felt that the health workers or staff did not listen and respond to their concerns, mainly in the West Bank 344/466 (73·7%). Few women reported not having a companion present at any time during labour and birth 53/745 (7·1%). 270/694 (38·9%) of women reported not having easy access to water and oral fluids during labour, and; only 30/535 (5·6%) were not allowed to eat.

Finally, the majority of women 69/745 (93·0%) reported having curtains, partitions or other measures for privacy.

Table 3 presents maternal interventions and newborn outcomes. Very few women reported perineal shaving 11/733 (1·5%), or edema 72/745 (9·7%). 140/700 (20·0%) of women reported having induction of labour, 194/745 (26·2%) reported having augmentation of labour. Most women with vaginal birth gave birth while laying on their back 462/542 (85·2%) and 77/542 (14·2%) gave birth while laying on back with legs separated. 722/745 (96·8%) of women had singleton births while 23/745 (3·2%) had a set of twins or triplets. Almost all babies were reported to be alive at birth. Only 6/576(1·04%) were stillbirth.

**Table 3:** Maternal and newborn interventions and health outcomes

	**Gaza** **(*N*=270)**	**West Bank** **(*N*=457)**	**Total** **(*N*=745)**
**Maternal interventions/ received**			
Induction of labour	49(20·1%)	91(20·0%)	140(20%)
Augmentation	68(25·4%)	126(26·6)	194(26·2%)
Enema	8(3·0%)	64.0(13·5%)	72(9·7%)***
Vaginal tear	103(38·0%)	168(35·4%)	271(36·4%)
Circumcision re-stitched	1(0·2%)	0(0·0%)	1(0·1%)
Shaving	10(3·7%)	1(2·0%)	11(1·5%)***
**Maternal Outcomes**			
Birth position *(vaginal birth only)			
Back\laying on back	187(92·6%)	275(80·9%)	462(85·2%)**
laying on back with legs separated	14(6·9%)	63(18·5%)	77(14·2%)
Others	1(0·5%)	2 (0·6%)	3 (0·6%)
**Newborn health outcomes**			
Singleton	262(97·0%)	460(96·8%)	722(96·9%)
Multiple	8(3·0%)	15(3·2%)	23(3·1%)
**Sex of the baby**			
Female	121 (44·8%)	174 (47·8%)	302(46·6%)
Male	146 (54·1%)	190 (52·2%)	334(52·9%)
Unknown	3 (1·1%	0	2(0·5%)
**Baby status at birth**			
Baby alive at birth	266 (98·5%)	310 (98·7%)	576(98·6%)
Stillbirth	3 (1·1%)	3 (0·9%)	6(1·0%)
Unknown/Refused to answer	1 (0·4%)	1 (0·3%)	2(0·3%)

^*^ *P*=0·05, ** *P*=0·01, *** *P*<0·0001

Table 4 presents the results of multivariate logistic regression models assessing factors potentially associated with mistreatment. Physical abuse was more likely to be reported by women with no companion with them (OR: 3·11, 95%CI: 1·24 − 7·99). Verbal abuse was more likely to be reported by women with less than three births (OR: 1·71, 95%CI: 1·06 − 2·76, women with no companion (OR: 2·72, 95%CI: 1·36 − 3·80) and more likely to be reported if curtains were not used (OR: 2·55, 95%CI: 1·33 − 4·88).

**Table 4:** Determinants of mistreatment of women during childbirth across five facilities in the oPt

**Item**	**Physical Abuse**	**Verbal Abuse**	**Non-Consented vaginal examination**	**No privacy or lack of privacy during vaginal examination**	**Long wait times or delays**	**Felt ignored, neglected or their presence was a nuisance**	**Mobilisation**
**Age**							
18-24	1·13 (0·33-3·88)	1·25 (0·71-2·22)	0·85 (0·55-1·34)	0·66 (0·32-1·36)	1·28 (0·83-1·98)	0·89 (0·54-1·46)	1·89 (0·86-4·16)
25-29	1·76 (0·56-5·47)	1·61 (0·97-2·72)	0·99 (0·65-1·51)	0·89 (0·46-1·73)	1·05 (0·70-1·59)	0·76 (0·48-1·20)	1·99 (0·99-4·01)
30+	1 (Ref)	1 (Ref)	1 (Ref)	1 (Ref)	1 (Ref)	1 (Ref)	1 (Ref)
**Education**							
Up to secondary education	1 (Ref)	1 (Ref)	1 (Ref)	1 (Ref)	1 (Ref)	1 (Ref)	1 (Ref)
Higher education	0·65 (0·27-1·54)	0·97 (0·63-1·48)	1·13 (0·80-1·59)	1·34 (0·77-2·34)	**1·40** (1·06-2·10)**	0·74 (0·51-1·08)	**0·52** (0·31-0·88)*
**Number of births**							
Up to 2 Births	1·78 (0·66-4·77)	**1·71** (1·06-2·76)*	**1·65** (1·10-2·45)*	0·98 (0·51-1·91)	1·31 (0·89-1·96)	0·54 (0·35-0·83)	0·98 (0·53-1·81)
3 births or more	1 (Ref)	1 (Ref)	1 (Ref)	1 (Ref)	1 (Ref)	1 (Ref)	1 (Ref)
**Curtains used**							
No	2·45 (0·68-8·71)	**2**·**55** **(1**·**33-4**·88)**	1·05 (0·56-1·98)	**3·94** (1·87-8·30)***	**3**·**29** **(1**·**83-5**·93)***	0·34 (0·19-0·62)	
Yes	1 (Ref)	1 (Ref)	1 (Ref)	1 (Ref)	1 (Ref)	1 (Ref)	1 (Ref)
**Companion presence at any time**							
No	**3·11** (1·24-7·99)*	**2·27** (1·36-3·80)**	1·50 (0·92-2·45)	0·717 (0·32-1·60)	0·82 (0·51-1·32)	0.71 (0·44-1·15)	1·17 (0·58-2·38)
Yes	1 (Ref)	1 (Ref)	1 (Ref)	1 (Ref)	1 (Ref)	1 (Ref)	1 (Ref)

^*^P=0·05, **P=0·01, ***P<0·0001

Not having consented vaginal examination was more likely to be reported by women with less than three births (OR: 1·65, 95%CI: 1·10 − 2·45). As expected, women were more likely to report lack of privacy during vaginal examination if curtains were not used (OR: 3·97, 95%CI: 1·87 − 8·30). Women were more likely to report long waiting time or delays in receiving services if they had lower education level compared to women with higher education (OR: 1·40, 95%CI: 1·06 − 2·10). There was an association between women reporting long waiting times and delays and curtains not being used (OR: 3·29, 95%CI: 1·83 − 5·93). Finally, women were less likely to report mobilization if they had higher education (OR: 0·52, 95%CI: 0·31 − 0·88).

The study was conducted during the COVID-19 pandemic. The most needed service by women were visiting the clinic 638/745 (85·6%) and medical consulting 531/745 (71·3%). Most women were able to visit the clinic 576/638 (90·2%); 489/531 (92·1%) and received medical consulting. One third of women 261/745 (35%) needed psychological support counseling and only 132/261 (51·0%) were able to receive this service. Low percent of women 197/745 (26%) needed information about Coronavirus and 129/197 (65·5%) of women received this information. One third of women 242/754 (32·5%) asked for gloves or/and masks while in the clinic or hospital and 200/242 (82·6%) received this service. 29/745 (3·9%) of women reported being infected with COVID-19 at the time of the interview and 83/745 (11·1%) had contact with a person infected with CVOID-19. Annex 5 presents reproductive health services needed and received by the participating women.

## Discussion

To our knowledge, this is one of the few studies measuring women’s experiences of care during childbirth in the Eastern Mediterranean region. In this study, we report women’s experiences during childbirth in five health facilities in the occupied Palestinian territory. The reported prevalence of any physical abuse, verbal abuse or stigma was 18·8%, with verbal abuse as the most common form of mistreatment compared to physical abuse and stigma and varied across Gaza and West Bank. Failure to meet professional standards, mainly non-consented procedures such as labour induction, episiotomy and cesarean birth, as well as vaginal examination, were commonly reported. Women were more likely to report different forms of mistreatment if they had less than three births, or where privacy measures were not used at the facility. Women were more likely to report positive experiences during their childbirth if they had a companion present at any time during labour and childbirth.

Compared to other findings, our findings on any physical abuse, verbal abuse or stigma and discrimination were lower than the range (48·3% in Nigeria to 20·8% in Myanmar) reported by women across four countries using the same community survey tool [16, 17]. Another study conducted post-partum interviews with women in the oPt and reported mistreatment during childbirth on vaginal examinations only [21]. According to Shallaby, the oPt women during birth were not always treated with respect and it was expected that their privacy was violated, with frequent lack of communication with health care providers and support from health care providers and family members [22]. While our study reports a prevalence of the mistreatment of women during childbirth using a validated community survey tool in the oPt, the comparability between studies is challenging as there are very few studies measuring this phenomenon in the region. Standardised measurements across the Eastern Mediterranean region are key to improving women’s experiences during childbirth and receiving respectful maternity care.

In the absence of a labour companion, women were more likely to report some forms of mistreatment, particularly physical and verbal abuse. Balde and colleagues also found that women in the absence of a labour companion experienced some, but not all, forms of mistreatment more often than women with labour companions [23]. It has also been shown that having a companion during labor and childbirth is associated with respectful care and can improve the experiences of women during labor and childbirth [24, 25]. An implementation research study conducted in the Eastern Mediterranean context found that the presence of birth companions improved women’s satisfaction during childbirth and was acceptable to health workers [5]. Studies have shown that labour companionship is highly context-dependent [23]. In our study context, there are differences between Gaza and the West Bank, where more women in Gaza reported different forms of mistreatment and lower levels of labour companionship. Another study showed that it is rare for birthing women to be supported during labour and birth by a family member in the Gaza strip [22] and therefore this may explain the differences in mistreatment between Gaza and West Bank. Overall, it is essential to sustain labour companions as part of continuous support in our context by ensuring an enabling facility environment (e.g. chair for the labour companion, privacy measures for women and their families) and maintaining positive health providers’ attitudes towards birth companions [26].

Our study highlighted failures in areas such as informed consent and communication. Health workers seem to marginalize the importance of obtaining a woman’s consent before any procedure and, lack communication skills such as listening and responding to women’s concerns. These failures might be explained partially by the high workload and shortage of staff, which force the health worker to work mechanically to complete the task. However, at the health system level, enabling environments to support health workers, including appropriate pre-service and in-service education/training, supervision and supportive policies, are critical to enable respectful care [27]. Specific to the Palestinian context, but which might apply to other conflict settings, is the effect of many years of continued political complexities, funding problems and no appropriate infrastructure resulting in weak and fragile health services. This has a direct effect on midwives and nurses who have to deal with many difficulties including low wages and limited social or professional guidance, which leads to providing substandard care [12]. The medicalization of the birthing process [28], and the objectification of women during the childbirth may also contribute to poor quality of care [11, 28, 29]. Furthermore, given the sensitive nature of procedures such as vaginal examinations, it is crucial to obtain informed consent and permission from the woman before conducting the examination and to clearly communicate the examination findings of [30]. Healthcare professional training, particularly on the quality of care related to provider–women communication, informed consented care, and ensuring women’s receipt of supportive care, are critical during the period of childbirth [28, 31].

Reports of mistreatment by Palestinian women were not mainly related to women’s characteristics, such as age or education. Contrary to the four-country study which reported a significant association between the different forms of mistreatment and women’s young age and lack of education [17], this association was not illustrated in the Palestinian context. In this context, women with higher education were more likely to know their rights and feel empowered to report long wait times to receive care.

This study was conducted during the COVID-19 pandemic. A global study reported that the COVID-19 pandemic has negatively affected the provision of respectful maternity care. This was explained by less family involvement, women receiving less emotional and physical support, compromised standards of care, increased unnecessary caesarean section and staff trying to cope with the rapidly changing guidelines and infection control measures [31]. Fortunately, although the health system in the oPt was struggling with the pandemic, it did not collapse. Quality of care in the Palestinian context, as in other countries, was compromised to some degree due to the factors listed above; however, the continued support of international agencies to most Arab countries ensured continuity on the provision of essential reproductive, maternal, neonatal, child and adolescent health services during the pandemic. This included training frontline healthcare workers, mainly midwives and nurses, on IPC measures in health facilities and establishing alternative delivery modalities for SRH services [32]. Our findings indicate that women could receive the needed services during the pandemic. Delivery in hospitals, antenatal care and postnatal care for high-risk pregnancies continued as usual during the pandemic while family planning services were scaled down [33]. Studies in Arab countries reported that fear of women infection or child infection were the main reason for avoiding antenatal visits during COVID-19. The service not being available due to the complete lockdown was another reason [34].

## Strengths and limitations

The study has unique strengths and limitations. First, it is the first study that documents mistreatment during childbirth in the Palestinian context using a standardized and validated tool. The tool was translated into the Arabic language following a robust methodology. Second, the study is based on a large sample size that included different regions of the oPt. Finally, the study was conducted during the COVID-19 pandemic, which helps understand the system functionality during this period. The study followed the master study conducted in the four countries with the exception of the data collection procedure [17]. The data collection was completed over the phone rather than through home visits which may have influenced reporting of mistreatment. The low cost of telephone surveys and their ability to reach larger participant samples assist in highlighting the scope of the issue.

While the interviews were conducted virtually, research from Nigeria has suggested that telephone interviews can be used to collect data on the experience of care; however, further research is needed to better understand changes over time, location and data collection method [35]. This change was decided to avoid personal contact and reduce the movement of data collectors between households due to the COVID-19 pandemic. Finally, the interviews were conducted with women after two to six weeks of childbirth, which might have introduced recall bias. One study in Tanzania reported disrespect and mistreatment more by women who were interviewed five to ten weeks after delivery [36]. Future studies should make an effort to speak with women more than once to comprehend the recall bias problem and gather thorough data. In addition to combining perceived and observed frequencies of mistreatment during childbirth.

## Conclusion

This is one of few studies informing the global and regional literature on women’s experiences of care in the Eastern Mediterranean region and specifically in a conflict setting such as the oPt. The results of this study are based on a standardized validated tool developed and tested in four countries. The tool was further translated, adapted and tested using a rigorous process in Arabic [37]. This community survey tool is a tool that covers almost all aspects of how women are treated throughout the birthing process that is related to the health systems, healthcare providers and their interactions with women and maternal outcomes. This tool can be used in other Arab countries to measure mistreatment during childbirth to allow regional comparison. For the first time using the WHO tool in the Eastern Mediterranean region, the study findings show the occurrence of mistreatment, mainly verbal abuse and non-consented procedures. In addition to identifying areas to be strengthened to ensure that all women have a respectful childbirth experience within health facilities. This can be achieved by introducing structural changes in childbirth models, such as supporting birth companions and improving health professional communication skills. In addition to building enabling environments to support health workers, including appropriate pre-service and in-service education/training, supervision and supportive policies.

## Supplementary Information

**Additional file 1: Annex 1. **Hospital Characteristics. **Annex 2.** Community Survey. **Annex 3.** Community survey: Physical abuse,verbal abuse, and stigma and discrimination. **Annex 4.** Community survey: Failure to meetprofessional standards of care, poor rapport between women and providers,health systems. **Annex 5.** Reproductive health services needed andreceived during COVID-19 pandemic. 

## Data Availability

All data generated or analyzed data during this study are included in this published article [Annex].
