# Supplementary material for: Exploring women’s experiences during childbirth in health facilities during COVID-19 pandemic in occupied palestinian territory: a cross-sectional community survey
Source: BMC Pregnancy Childbirth. 2022 Dec 22;22:957. doi: 10.1186/s12884-022-05265-y (PMC9773592; doi:10.1186/s12884-022-05265-y)
Supplement: Supplementary file 1 — Additional file 1: Annex 1. Hospital Characteristics. Annex 2. Community Survey. Annex 3. Community survey: Physical abuse,verbal abuse, and stigma and discrimination. Annex 4. Community survey: Failure to meetprofessional standards of care, poor rapport between women and providers,health systems. Annex 5. Reproductive health services needed andreceived during COVID-19 pandemic. [file 12884_2022_5265_MOESM1_ESM.docx]

Annex 1: Hospital Characteristics

| **Region** | **Hospital 1**  **(Ramallah)** | **Hospital 2**  **(Ramallah)** | **Hospital 3**  **(Hebron)** | **Hospital 4**  **(Hebron)** | **Hospital 5**  **(Gaza)** |
| --- | --- | --- | --- | --- | --- |
| Locality | urban | urban | urban | urban | urban |
| Management | public | private | public | private | public |
| Cost of vaginal birth (USD) | $30-212, depending on insurance coverage | $80-700, depending on insurance coverage | $30-212, depending on insurance coverage | $80-700, depending on insurance coverage | *** |
| Cost of caesarean birth (USD) | $90-303, depending on insurance coverage | $120-1550, depending on insurance coverage | $90-303, depending on insurance coverage | $100-760, depending on insurance coverage | *** |
| Total births per month | 400-500 | 150-200 | 600-700 | 450-500 | 1500-1700 |
| Number of beds - obstetrics | 13 | 15-18 | 37 | 22 | *** |
| Caesarean section rate |  | 42% | 15% |  |  |
| # obstetricians per shift | 3 | 4 (upon request) | 5-6 | 3 (upon request) |  |
| # medical officers/junior doctors per shift | 5 | **** | 7-8 |  |  |
| # midwives per shift | 6-8 | 5 | 7-9 | 10-12 |  |
| Estimated population of the catchment area** | 200000 | 200000 | 300000 | 300000 |  |
| Facility-based childbirth in catchment area | *** | *** | 55-60% | 15-16% |  |

Annex 2: Community Survey

|  | **West Bank** | | **Gaza** | |
| --- | --- | --- | --- | --- |
|  | N | % | N | % |
| Overall sample | 475 | 63·7 | 270 | 36·3 |
| Number of children alive today |  |  |  |  |
| 1 | 118 | 24·8 | 84 | 31·1 |
| 2 | 125 | 26·3 | 49 | 18·1 |
| 3 | 98 | 20·6 | 45 | 16·6 |
| 4+ | 134 | 28·2 | 92 | 34·1 |
| Currently breastfeeding |  |  |  |  |
| YES | 434 | 91·3 | 259 | 95·9 |
| NO | 41 | 8·7 | 11 | 4·07 |
| Breastfeeding initiation |  |  |  |  |
| < 1 hour | 287 | 60·4 | 163 | 60·4 |
| < 24 hours | 110 | 23·1 | 77 | 28·5 |
| One week | 31 | 6·5 | 15 | 5·6 |
| Longer than one week | 6 | 1·2 | 4 | 1·5 |
| Baby Status | merge |  |  |  |
| Alive |  |  |  |  |
| Stillbirth |  |  |  |  |
| Child alive when he/she was born |  |  |  |  |
| Child alive when you left the hospital |  |  |  |  |

Annex 3: Community survey: Physical abuse, verbal abuse, and stigma and discrimination

|  | **West Bank** | | **Gaza** | |
| --- | --- | --- | --- | --- |
|  | **N** | **%** | **N** | **%** |
| **Overall sample** | 475 | 63·7 | 270 | 36·3 |
| **Any physical abuse, verbal abuse, or stigma and discrimination** | 78 | 16·4 | 62 | 22·9 |
| **Any physical abuse** | 11 | 2·3 | 14 | 5·2 |
| Slap | 1 | 0·2 | 0 | 0 |
| Punch | 0 | 0 | 0 | 0 |
| Beat | 0 | 0 | 0 | 0 |
| Put something in the patient mouth | 2 | 0·42 | 0 | 0 |
| Tied to the bed | 0 | 0 | 0 | 0 |
| Held down to the bed forcefully | 1 | 0·2 | 0 | 0 |
| Forcefully placed pressure on your abdominal area before giving birth? | 6 | 1·2 | 13 | 4·8 |
| Other physical | 2 | 0·6 | 0 | 0 |
| **Any verbal abuse** | 70 | 14·7 | 54 | 20·0 |
| Scream | 33 | 6·9 | 32 | 6·7 |
| Insult | 9 | 1·9 | 4 | 1·5 |
| Tell you off | 19 | 4·0 | 10 | 1·3 |
| Make fun of you | 9 | 1·9 | 2 | 0·74 |
| Comment negatively on your physical appearance | 0 | 0 | 2 | 0·74 |
| Comment negatively on your child’s appearance | 1 | 0·2 | 1 | 0·37 |
| Comment negatively regarding your sexual activity | 0 | 0 | 0 | 0 |
| Workers threaten you with a medical procedure | 8 | 1·7 | 5 | 1·9 |
| Workers threaten you with physical violence? | 0 | 0 | 2 | 0·74 |
| Threaten you with having or your child having poor outcomes | 21 | 4·4 | 13 | 4·8 |
| Threaten you with withholding or stopping care for you or your child? | 2 | 0·4 | 2 | 0·74 |
| Blame | 14 | 5·19 | 6 | 2·2 |
| Workers try to silence you by making a sound | 20 | 4·2 | 24 | 8·9 |
| Other form of verbal abuse used against you | 3 | 0·6 | 1 | 0·4 |
| **Any stigma and discrimination** | 4 | 0·8 | 1 | 0·4 |
| Comment negatively about your ethnic origin | 1 | 0·2 | 0 | 0 |
| Workers comment negatively about your religion | 0 | 0 | 0 | 0 |
| Comment negatively about your age | 3 | 0·6 | 1 | 0·4 |
| Comment negatively about whether you are married | 0 | 0 | 0 | 0 |
| Level of education | 0 | 0 | 0 | 0 |
| Comment negatively about your economic status | 0 | 0 | 0 | 0 |
| Comment negatively about your HIV | 0 | 0 | 0 | 0 |

Annex 4: Community survey: Failure to meet professional standards of care, poor rapport between women and providers, health systems

|  | **West bank** | | **Gaza** | |
| --- | --- | --- | --- | --- |
|  | **N** | **%** | **N** | **%** |
| **Informed consent and confidentiality** |  |  |  |  |
| Was the procedure explained and did you agree to the procedure? |  |  |  |  |
| *Caesarean section* N=745 | 135 | 28·4 | 68 | 25·2 |
| IF SELECTION by= CS |  |  |  |  |
| Not explained, did not agree | 1 | 0·7 | 0 | 0 |
| Not explained, agreed | 42 | 31·0 | 24 | 35.3 |
| Explained, did not agree | 92 | 68·3 | 42 | 61·8 |
| Explained and agreed | 0 | 0 | 2 | 2·9 |
| *Episiotomy* N= 251 | 148 | 57·5 | 103 | 38·0 |
| Not explained, did not agree | 1 | 0·6 | 18 | 17·4 |
| Not explained, agreed | 42 | 28·4 | 33 | 32·1 |
| Explained, did not agree | 105 | 70·9 | 41 | 39·9 |
| Explained and agreed | o | 0 | 11 | 10·6 |
| **Vaginal examinations** |  |  |  |  |
| Woman had any vaginal examination? **yes** | 402 | 84·6 | 228 | 84·0 |
| Non-consented vaginal examination | 269 | 66·9 | 118 | 51·7 |
| Vaginal examination conducted privately ? **yes** | 369 | 91·8 | 193 | 84·6 |
| Discuss your private information privately? **yes** | 331 | 82·5 | 162 | 71·7 |
| *General description of experience of vaginal examinations* | 91 | 22·7 | 87 | 38·2 |
| Comfortable | 157 | 38·9 | 91 | 39·9 |
| A little uncomfortable | 99 | 24·7 | 35 | 15·4 |
| Quite uncomfortable | 55 | 13·7 | 15 | 6·6 |
| Very uncomfortable | 91 | 22·7 | 87 | 38·2 |
| **Pain relief** |  |  |  |  |
| Woman not offered pain relief during time in hospital | 280 | 59·2 | 198 | 73·6 |
| Woman requested pain relief | 274 | 57·8 | 110 | 40·7 |
| Woman did not receive pain relief | 109 | 39·8 | 98 | 89·1 |
| Woman denied pain relief during time in hospital | 53 | 11·2 | 59 | 22·3 |
| **Neglect and abandonment** |  |  |  |  |
| Was a staff member present when the baby came out? (among n=542 vaginal births) |  |  |  |  |
| **no** | 40 | 7·3 | 15 | 2·8 |
| **yes** | 295 | 86·7 | 182 | 90·5 |
| **Refuse** | 5 | 0·9 | 4 | 0·7 |
| Woman waited for long periods of time before attended by health workers? **Yes** | 141 | 29·7 | 101 | 38·1 |
| Woman felt ignored, neglected, or that presence was a nuisance for health workers or staff? **yes** | 88 | 18·6 | 86 | 31·8 |
| Woman felt that health workers or staff did not listen and respond to her concert? **Agree** | 344 | 73·7 | 144 | 54·5 |
| **Supportive care** |  |  |  |  |
| Woman not allowed to have labour companion during labour process | 38 | 8·0 | 60 | 22·3 |
| Companion not present at any time and birth | 44 | 9·3 | 9 | 3·3 |
| **Autonomy** |  |  |  |  |
| Woman did not have easy access to water or oral fluids during labour | 149 | 33·5 | 121 | 48·6 |
| Not allowed to eat (women with vaginal birth) | 21 | 6·2 | 9 | 4·5 |
| Woman not told she could mobilise during labour, and did not mobilise during labour | 115 | 24·2 | 105 | 39·2 |
| Woman not allowed to deliver in her preferred position | 8 | 35·3 | 2 | 12·5 |
| Woman or baby detained in hospital because of inability to pay hospital bills | 1 | 0·2 | 2 | 0·7 |
| Actual birth position (for women with vaginal birth, n=542) |  |  |  |  |
| Back /Lying on back | 275 | 80·9 | 187 | 92·6 |
| Lying on your back with legs separated and suspended | 63 | 18·5 | 14 | 6·9 |
| Sitting | 0 | 0 | 1 | 0·5 |
| Lying on her side | 1 | 0·3 | 0 | 0 |
| other | 1 | 0·3 | 0 | 0 |
| **Health systems** |  |  |  |  |
| Woman instructed to clean up blood, urine, faeces, or amniotic fluid | 28 | 5·7 | 8 | 2·6 |
| Staff suggested or asked the woman or companion for a bribe, informal payment, or gift* | 9 | 1·9 | 160 | 59·7 |
| Used Curtains, partitions, or other measures used to provide privacy for the woman throughout labour, childbirth, and post-partum periods | 440 | 92·6 | 251 | 93·7 |

**Annex 5:** Reproductive health services needed and received during COVID-19 pandemic

|  | **Gaza**  **(N=270)** | **West Bank**  **(N=457)** | **Total**  **(N=745)** |
| --- | --- | --- | --- |
| **Visit the clinic** |  |  |  |
| Did you need this service? | 219(81·1%) | 419(88·2%) | 638(85·6%)** |
| Did you receive this service? | 197(90·0%) | 379(90·5%) | 576(90·2%)* |
| **Medical consulting** |  |  |  |
| Did you need this service? | 190(70·4%) | 341(71·8%) | 531(71·3%) |
| Did you receive this service? | 179(94·2%) | 310(90·9%) | 489(92·1%) |
| **Psychological support counseling** |  |  |  |
| Did you need this service? | 120(44·4%) | 141(29·7%) | 261(35·0%)*** |
| Did you receive this service? | 67·0(55·8%) | 65(46·8%) | 132(51·0%)*** |
| **Provide information about the COVID-19** |  |  |  |
| Did you need this service? | 88·0(32·6%) | 109(22·9%) | 197(26·4%)** |
| Did you receive this service? | 62·0(70·5%) | 67·0(61·5%) | 129(65·5%)** |
| **Provide information about ways to prevent the virus at childbirth / in the hospital** |  |  |  |
| Did you need this service? | 74·0(27·4%) | 69·0(14·5%) | 143(19·2%)*** |
| Did you receive this service? | 48·0(64·84%) | 28·0(40·6%) | 67(46·8%)*** |
| **Providing means of prevention (gloves and masks) in the clinic / in the hospital** |  |  |  |
| Did you need this service? | 68(25·2%) | 174(36·6%) | 242(32·5%)** |
| Did you receive this service? | 43 (63·2%) | 157(90·2%) | 200(82·6%)*** |
| **Are you infected with COVID-19?** | 2 (0·7%) | 27 (5·7%) | 29 (3·9%)** |
| **Do you know or have had contact with a person infected with COVID-19?** | 2 (0·7%) | 81 (17·1%) | 83 (11·1%)*** |

* P=0·05, ** P=0·01, *** P<0·0001
